# The Relationship Between C-reactive Protein Albumin Ratio and Long-Term Mortality in Patients With Acute Coronary Syndrome

**DOI:** 10.7759/cureus.47222

**Published:** 2023-10-17

**Authors:** Oğuzhan Birdal

**Affiliations:** 1 Cardiology, Ataturk University Faculty of Medicine, Erzurum, TUR

**Keywords:** long term mortality, acute coronary syndrom, crp albumin ratio, albumin, crp, inflammation

## Abstract

Background

Coronary artery disease (CAD) is one of the leading causes of death worldwide. CRP/albumin ratio is a sensitive indicator of inflammatory status. It has been shown that this parameter may be associated with poor short-term outcomes in CAD. In this study, we investigated the relationship between long-term mortality and the CRP/albumin ratio in patients with acute coronary syndromes (ACS).

Material and methods

This study was conducted on patients who applied to our hospital between January 2015 and December 2019 and were diagnosed with ACS. A total of 1689 patients were included in the study. Patients were divided into two groups according to mortality status, and long-term mortality predictors were investigated.

Results

The average follow-up period was 38.9±10.3 months. The mean age of the entire study group was 56.6±12.2 years, and 1440 (80.5%) of the patients were male. Comorbid diseases and blood parameters were significant between the two groups. In the regression analysis, creatine, hemoglobin, white blood cell count (WBC), neutrophil-lymphocyte ratio (NLR), and CRP albumin ratio (CAR) were found to be independent predictors. In the ROC analysis, it was observed that CAR had the best predictive value.

Discussion

An increased CAR level is an independent predictor of long-term mortality in ACS patients. It can be used in both short-term and long-term risk stratification for ACS patients.

## Introduction

Cardiovascular diseases (CVD) are the most common cause of death worldwide. Coronary artery disease (CAD) is the most important disease in this disease spectrum. CAD develops with the reduction of blood flow to the myocardium due to the deterioration of the coronary vessels for various reasons. Atherosclerosis is the most important of these causes. Atherole plaque forms as a result of atherosclerosis. As a result of rupture or erosion of the atheroma plaque, acute coronary syndrome (ACS) develops [1]. This clinic may result in unstable angina (UAP), non-ST-elevation myocardial infarction (NSTEMI), ST-elevation myocardial infarction (STEMI), and sudden cardiac death [2]. Mortality and morbidity have decreased due to pharmacological treatments and improved treatment methods in recent years, but deaths still continue to be an important health problem.

Inflammatory pathways play a major role in the formation and progression of atherosclerosis, the most common cause of CAD [3]. Many markers are used in clinical practice to detect inflammation. Hemogram parameters, albumin, and C-reactive protein (CRP) are the most frequently used parameters. Studies have shown that these markers can be used to predict cardiac risk and guide treatment [4-7]. CRP is a positive acute phase reactant, and albumin is a negative acute phase reactant. Therefore, it has been thought that the CRP/albumin ratio (CAR) may be a more sensitive indicator of the severity of the inflammatory reaction and disease progression in various patient groups [8,9]. Studies have shown that there is a significant relationship between CAR and CAD in the short term [10]. The aim of our study was to investigate the relationship between inflammatory parameters and long-term mortality in ACS patients.

## Materials and methods

Study design

Patients applied to the Emergency Service and Cardiology Clinic of Atatürk University Faculty of Medicine Research Hospital between 2015 and 2019, and patients who were diagnosed with ACS and underwent coronary angiography (CAG) were included in the study. The institutional review board of our hospital approved the study protocol on December 07, 2023 (number: B.30.2.ATA.0.01.00/593). All procedures were applied in accordance with the 2013 update of the Declaration of Helsinki.

Before the coronary angiography procedure, blood was taken from the peripheral vein for biochemical and hematological tests. CAG images were viewed by two different cardiologists unaware of each other, and their measurements were recorded. In addition, the patient's past disease information, physical examination findings, blood tests, electrocardiography (ECG), and echocardiography (ECHO) data were obtained from hospital records. Patients with inappropriate medical records, patients with active infection, malnutrition, active inflammatory disease states, and malignancy, and patients whose archive records could not be accessed due to technical reasons, were excluded from the study.

Statistical analysis

All statistical studies were analyzed with SPSS Statistics for Windows, Version 22.0 (SPSS Inc., Chicago, IL, USA). Percentages were used to show categorical variables, and continuous variables were presented as mean ± standard deviation or median (interquartile range) according to the fit for a normal distribution. Parametric variables belonging to two independent groups were evaluated with the t-test, and categorical variables were evaluated with the appropriate chi-square test. The Mann-Whitney U test was used to analyze the variables of two groups that did not comply with a normal distribution. Cox regression analysis was used to find independent predictors of mortality development. Parameters that were significant in univariate analysis were evaluated with correlation analysis, and then multivariate analysis was performed by modeling. ROC curve analysis was performed to evaluate the sensitivity and specificity values of the parameters in mortality prediction. Variables with a P-value <0.05 were considered statistically significant.

## Results

A total of 1689 patients were included in the study. The average follow-up period of the patients was 38.9±10.3 months. The mean age of the entire cohort was 56.6±12.2 years, and 1440 (80.5%) of the patients were male. While the average age in the mortality group was 65.8±12.8 and 67.8% of the patients were male, in the survival group, the average age was 55.5±11.7 and 82% of the patients were male (p<0.001 for both parameters). When comorbid diseases were examined, a statistically significant difference was observed between the two groups. In the mortality group, HT, DM, COPD, and previous MI were higher, and the EF of the patients was lower (p<0.001 for each parameter). Additionally, when laboratory parameters were evaluated, it was observed that both hemogram parameters and inflammation parameters were significant between the two groups. Basal characteristic features are given in Table 1.

**Table 1:** Basal characteristics of the groups. HT: hypertension, DM: diabetes mellitus, MI: miyocardial infarction, COPD: chronic obstructive pulmonary disease, EF: ejection fraction, WBC: white blood cell, NLR: neutrophil lymphocyte ratio, CRP: C-reactive protein, CAR: CRP albumin ratio, LDL: low-density lipoprotein, HDL: high density lipoprotein. Data hve been represented as numbers (n) and percentages (%), and mean ± SD (standard deviation) or median (interquartile range 25-75) where mentioned. Significant p-value were defined if less than 0.05.

Variables	Mortality (n=180)	Survival (n=1509)	p-value
Age (year)	65.8±12.8	55.5±11.7	<0.001
Gender (male,%)	122 (67.8)	1237 (82)	<0.001
HT (n,%)	98 (54.4)	584 (38.7)	<0.001
DM (n,%)	82 (45.6)	302 (20)	<0.001
Previous MI (n,%)	9 (5)	16 (1.1)	<0.001
COPD (n,%)	18 (10)	65 (4.3)	<0.001
EF (%)	38.8±9.1	48.6±7.3	<0.001
Glucose (mg/dL)	200±108	142±67	<0.001
Creatine (mg/dL)	1.30±0.8	0.89±0.4	<0.001
Hemoglobin (g/dL)	12.7±2.2	13.8±1.7	<0.001
WBC (10^3^/µL)	14.5±5.5	11.9±3.3	<0.001
Neutrophil (10^3^/µL)	11.8±4.9	9.1±3.2	<0.001
Lymphocyte (10^3^/µL)	1.70±0.87	2.01±1.04	<0.001
NLR	7.39 (4.67–11.69)	4.81 (3.11–7.33)	<0.001
Platelet (10^3^/µL)	262.2±74.8	256.6±65.3	0.261
Albumin (g/dL)	3.43±0.53	3.79±0.47	<0.001
CRP (mg/L)	21.1 (12.1–34.2)	9.1 (5.3–15.4)	<0.001
CAR	5.86 (3.26–10.68)	2.41 (1.32–4.16)	<0.001
Troponin I (ng/mL)	3.45 (1.40–11)	1.78 (0.65–4.24)	<0.001
Total cholesterol (mg/dL)	167.8±47.3	179.2±42.8	0.001
LDL cholesterol (mg/dL)	105.6±39.7	114.3±37.9	0.002
HDL cholesterol (mg/dL)	38.5±15.1	39.1±11.8	0.070
Triglyceride	126.6±57.1	140.1±92.9	0.544

Parameters that were significant in the univariate analysis were included in the correlation analysis. High and very high correlation parameters were not included in the regression analysis together. As a result of Cox regression analysis, creatine (p<0.001), hemoglobin (p<0.001), WBC (p=0.006), NLR (p=0.016), and CAR (p<0.001) were found to be independent predictors of long-term mortality. Univariate and multivariate analysis results are shown in Table 2.

**Table 2:** Regression analysis of blood parameters according to mortality WBC: white blood cell, NLR: neutrophil lymphocyte ratio, CAR: CRP albumin ratio, LDL: low-density lipoprotein. Data have been represented as numbers (n) and percentages (%), and mean ± SD (standard deviation) or median (interquartile range 25-75) where mentioned. Significant p-value were defined if less than 0.05.

Variables	Univariate OR, 95 CI%	P-value	Multivariate OR, 95 CI%	P-value
Glucose	1.04 (1.03–1.06)	<0.001	1.01 (1–1.02)	0.093
Creatinine	1.66 (1.43–1.93)	<0.001	1.56 (1.25–1.95)	<0.001
Hemoglobin	0.76 (0.73–0.84)	<0.001	0.80 (0.71–0.90)	<0.001
WBC	1.08 (1.03–1.13)	<0.001	1.08 (1.02–1.15)	0.006
NLR	1.05 (1.03–1.07)	<0.001	1.04 (1.01–1.07)	0.016
CAR	1.19 (1.15–1.24)	<0.001	1.17 (1.12–1.21)	<0.001
Troponin I	1.06 (1.04–1.07)	<0.001	1.01 (0.98–1.04)	0.702
LDL cholesterol	0.94 (0.86–0.98)	<0.001	0.99 (0.98–1.01)	0.447

**Figure 1:**
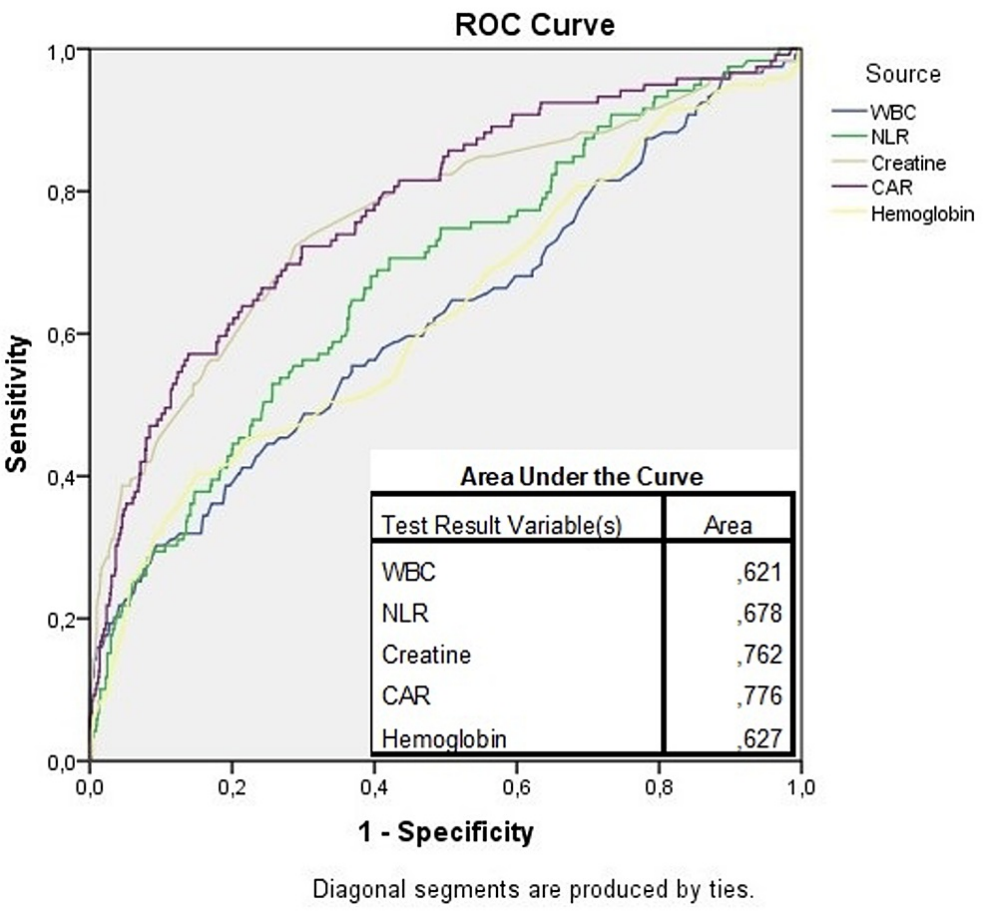
ROC curve of the parameters that were significant in the regression analysis (to avoid visual misunderstanding, the hemoglobin curve is given as n-1).

ROC analysis was performed to determine the parameter with the best predictive value among the parameters that were significant in the Cox regression analysis. As a result of ROC analysis, it was determined that CAR had the largest area under the curve and thus had the best predictive value among independent predictors. ROC curve analysis results are given in Figure 1.

## Discussion

This study showed that CAR was an independent predictor of long-term mortality as well as short-term mortality, as shown in other studies [10].

CAD prevalence increases with aging. The lifetime risk of developing CAD in men and women after 40 years of age is 49% and 32%, respectively [11]. The increase in comorbid diseases with aging, the increase in fragility, and the insufficient access to health opportunities cause mortality [12]. In addition to age, gender differences are important in the diagnosis and follow-up of CAD patients [13]. CVD develops approximately 10 years later in women than in men. Therefore, the risk of CVD in women is often underestimated due to the misperception that females are protected against CVD. Although the incidence of CVD in women is usually lower than in men, women have a higher mortality rate and a worse prognosis after acute cardiovascular events [14]. Vogel et al. have found that women with STEMI have a worse prognosis during their hospital stay compared to men [15]. In the present study, age and gender differences were observed between the groups. Consistent with the literature, the mortality group was older, and the number of female patients was higher.

Comorbid diseases are very common in patients with CVD, even in younger age groups [16]. Comorbid diseases such as HT, DM, and COPD, which are the most common in CAD patients, are also chronic diseases, and their prevalence is gradually increasing [16]. In addition to the problems caused by CAD, these comorbid diseases impair the quality of life and cause morbidity and mortality [17]. In this study, it was observed that comorbid diseases were significantly more common in the mortality group.

The effect of inflammation on atherosclerosis development and destabilization has been more clearly understood in recent years, and inflammatory biomarkers are now increasingly being used in CAD screening and prognosis. One of the most easily accessible inflammation parameters is white blood cell count (WBC), which is one of the hemogram parameters. Studies have shown that there is a relationship between WBC and CAD [18]. In addition, the neutrophil-lymphocyte ratio (NLR) derived from WBC parameters is also used in CAD [19,20]. Kaya et al. showed that there was a significant relationship between CAD severity and NLR [21]. Therefore, Angkananard et al. demonstrated that high NLR was associated with CAD, ACS, stroke, and composite cardiovascular events [22]. In our study, similar to these studies, WBC and NLR were found to be associated with CAD and were found to be independent predictors of long-term mortality.

One of the hemogram parameters is the patient's hemoglobin level. Having anemia in the patient can mimic the symptoms of CAD, yet anemia is also associated with CAD [23]. Similarly, decreased hemoglobin level was found to be an independent predictor of long-term mortality in our study.

Serum creatine level is an indicator of kidney function. Kidney functions are important for cardiac functions [24]. It is also a parameter that should be considered in pharmacological treatment. Increased serum creatine levels are associated not only with CAD but also with CVD [25]. The presence of chronic renal failure (CRF) is a major risk factor for developing CAD [26]. Korkmaz et al. showed that creatine level correlated with CAD and was associated with the severity of CAD [27]. In our study, creatine levels were found to be associated with mortality, similar to the literature.

Albumin level is a cardiovascular prognostic biomarker. It has been shown that a lower albumin level is related to coronary artery disease severity and poor outcomes in CAD [4]. In addition, CRP, one of the most commonly used biomarkers for demonstrating inflammation, has been associated with coronary events, the severity of CAD, and cardiac mortality in patients with CAD [28]. Fairclough et al. described CAR as a more accurate prognostic indicator than either serum CRP or albumin levels when it comes to predicting a poor prognosis in patients with acute medical conditions [29]. CAR is more sensitive and specific in predicting the systemic inflammatory state and prognosis in many clinical situations when compared to CRP and serum albumin separately [9,10]. Most studies about CAR include in-hospital events and short-term follow-ups. In the present study, we showed that there is a significant relationship between mortality and CAR in the long-term follow-up of patients with ACS and found that CAR is an independent predictor of mortality.

This study has some limitations. First, our study was performed in a single center and had a retrospective design. Due to the retrospective design, there may also be selection bias due to unobserved variables and missing variables. Second, we do not have data on why the patients died, and we do not have data on the medications the patients used. Third, since acute coronary syndrome is a broad spectrum, our cohort had a heterogeneous patient distribution, and baseline characteristics were different.

## Conclusions

Inflammation parameters are used in the diagnosis, treatment, and prognosis of many diseases and show their effectiveness. Considering they are easily accessible, cost-effective, and simple to interpret, inflammatory parameters play an important role in disease evaluation.

A higher CAR level at hospitalization was found to be associated with long-term mortality in ACS patients. In these patients, CAR has been found to be a more precise marker than the use of other inflammatory parameters alone. Therefore, CAR can be used in ACS patients to predict poor outcomes and to monitor the patient more closely.
